# Validity of Center of Pressure Path Length Measured Using a Wii Balance Board for Fall Risk Screening in Community-Dwelling Older Adults

**DOI:** 10.3390/healthcare14121685

**Published:** 2026-06-12

**Authors:** Myeong-Min Ju, Dae-Sung Park

**Affiliations:** Department of Physical Therapy, Konyang University, Daejeon 35365, Republic of Korea; 24853502@konyang.ac.kr

**Keywords:** falls, balance assessment, center of pressure, Wii Balance Board, older adults, posturography

## Abstract

**Highlights:**

**What are the main findings?**

- Center of pressure (COP) path length measured using a Wii Balance Board showed significant associations with the Tetrax^®^ Fall Index and conventional functional performance measures.
- COP path length measured under the normal eyes-open condition demonstrated the highest discriminatory performance among the tested sensory postural conditions.

**What are the implications of the main findings?**

- A simple and low-cost balance assessment using a portable device may provide a feasible approach for balance assessment in community-dwelling older adults.
- These findings support the exploratory feasibility of simplified Wii Balance Board–based balance assessment approaches in community and primary healthcare settings.

**Abstract:**

**Background/Objectives**: Falls among older adults are a major public health concern. Although instrumented posturography provides objective balance and fall-risk assessment, its cost and limited portability restrict widespread use. This study aimed to examine the construct and concurrent validity of center of pressure (COP) path length measured using a Wii Balance Board (WBB) in relation to a clinically established posturographic fall-risk construct in community-dwelling older adults and to explore its discriminatory performance across multiple sensory postural conditions. **Methods**: Sixty adults aged ≥ 65 years participated in this cross-sectional study. COP path length was measured using a WBB under eight postural conditions and compared with the Fall Index derived from a conventional posturography system (Tetrax^®^). Functional performance was assessed using the Four Square Step Test and the Five Times Sit-to-Stand test. Pearson correlation, receiver operating characteristic (ROC), and exploratory regression analyses were performed. **Results**: COP path length showed significant positive correlations with the Tetrax^®^ Fall Index across all conditions (r = 0.349–0.561, *p* < 0.01) and with functional performance tests under most postural conditions (*p* < 0.05), except for the Normal stability, Open eyes (NO) condition. ROC analysis demonstrated acceptable-to-good discriminatory performance for classifying Tetrax^®^ Fall Index-based risk status (AUC = 0.783–0.865), with the NO condition showing the highest discriminatory capability (AUC = 0.865). Exploratory regression models based on selected postural conditions explained 12.1–40.7% of the variance in the reference Fall Index. **Conclusions**: COP path length measured using a WBB demonstrated construct validity and acceptable discriminatory capacity in relation to a conventional posturographic fall-risk construct in community-dwelling older adults. These findings support the exploratory feasibility of simplified WBB-based balance assessment approaches for community and clinical screening contexts. Further longitudinal studies incorporating prospective fall outcomes are required to establish predictive validity and broader clinical applicability.

## 1. Introduction

With the rapid growth of the aging population, falls have emerged as a major public health concern among older adults, contributing to functional decline, reduced quality of life, and increased healthcare costs [1]. Falls are closely associated with serious consequences such as fractures, hospitalization, long-term care dependency, and increased mortality [2]. Accordingly, the early identification of fall risk and the implementation of effective preventive strategies are critical issues in both clinical practice and community-based healthcare settings [3].

Several functional performance tests, including the Five Times Sit-to-Stand Test (5× STS) and the Four Square Step Test (FSST), are commonly used to assess fall risk in older adults [4,5]. These tests are easy to administer and require minimal equipment; however, their ability to capture subtle balance impairments is limited. Moreover, test outcomes may be influenced by examiner skill, and the lack of objective quantitative measures restricts their usefulness for precise fall-risk classification [6]. In contrast, instrumented balance assessment systems such as the Tetrax^®^ balance system provide detailed postural stability data but are often costly, non-portable, and impractical for widespread use in clinical and community environments [7].

In recent years, the Wii Balance Board (WBB) has gained attention as a low-cost and portable alternative for balance assessment. By measuring center of pressure (COP) parameters, the WBB enables quantitative evaluation of postural control and has demonstrated acceptable reliability and validity in previous studies [8]. Its affordability and portability suggest considerable potential for use beyond laboratory settings. Nevertheless, most previous studies have primarily examined the concurrent validity of WBB-derived COP parameters against laboratory-grade force plates, with limited investigation of whether WBB-based measures can approximate clinically established multidimensional fall-risk constructs derived from validated posturographic systems [9].

Because postural control in older adults depends on the integration of visual, vestibular, and somatosensory systems, balance impairments may manifest differently according to sensory challenge conditions [10]. Therefore, evaluating COP behavior across multiple standardized postural conditions may provide a more comprehensive representation of balance-related fall susceptibility than single-condition assessment alone [11]. In particular, the present study examined eight standardized postural conditions that systematically modulated visual, vestibular, and somatosensory inputs, thereby enabling condition-specific assessment of sensory integration and its relationship with clinically validated fall-risk measures. Unlike previous WBB studies that examined a limited number of standing conditions, this multi-condition approach may contribute to a more comprehensive understanding of how condition-specific COP responses relate to fall-risk characteristics in older adults.

In addition, although WBB-derived COP parameters have demonstrated potential for balance assessment, the feasibility of developing a portable WBB-based fall-risk estimation framework has not been sufficiently explored [12,13]. Rather than directly reproducing proprietary fall-risk scoring systems, exploratory modeling approaches using multiple sensory-condition COP measures may provide preliminary insight into the development of accessible and clinically applicable fall-risk estimation tools. Furthermore, the relationships between WBB-derived measures and conventional functional performance tests remain incompletely understood.

Therefore, the primary purpose of this study was to examine the construct and concurrent validity of COP path length measured using a WBB by comparing it with a validated posturography-derived Fall Index and functional performance measures in community-dwelling older adults. A secondary exploratory objective was to investigate the feasibility of developing a prototype WBB-derived fall-risk estimation framework based on multiple sensory postural conditions. Additionally, receiver operating characteristic (ROC) analysis was conducted to determine clinically relevant cut-off values for fall-risk classification.

## 2. Materials and Methods

### 2.1. Study Design

This study employed a cross-sectional design. The primary aim was to examine the construct and concurrent validity of center of pressure (COP) path length measured using a Wii Balance Board (WBB) in community-dwelling older adults aged 65 years or older. Associations between COP path length and the Fall Index derived from the Tetrax^®^ system (Sunlight Medical Ltd., Ramat Gan, Israel), the Four Square Step Test (FSST), and the Five Times Sit-to-Stand test (5× STS) were analyzed. A secondary exploratory aim was to investigate the feasibility of developing a prototype WBB-derived fall-risk estimation framework using multiple sensory postural conditions. In addition, receiver operating characteristic (ROC) analysis was conducted using the Tetrax^®^ Fall Index classification as the reference criterion to determine COP path length cut-off values for fall-risk classification.

### 2.2. Participants

Sixty older adults (15 men and 45 women) aged 65 years or older participated in this study. All participants received a detailed explanation of the study purpose and procedures and provided written informed consent prior to participation. Inclusion criteria were the ability to independently perform sit-to-stand movements and ambulation without assistive devices, and no history of pelvic or lower-extremity fractures within the previous year. Exclusion criteria included the presence of lower-extremity orthopedic conditions (e.g., rheumatoid arthritis, cartilage or ligament injuries), inability to understand study instructions or communicate effectively, and current use of medications known to affect postural stability, balance, or alertness, as medication-related factors may influence fall risk and postural control in older adults [14,15]. This study was conducted with the approval of the Institutional Review Board (IRB) of K University (KYU-2023-06-021-002).

The required sample size was determined a priori using G*Power (version 3.1.9.7; Heinrich Heine Universität Düsseldorf, Düsseldorf, Germany). Because the primary objective of the study was validity assessment through correlation analyses between WBB-derived COP measures and established fall-related measures, the sample size calculation was based on Pearson correlation analysis. Based on a medium correlation effect size (ρ = 0.35), a two-tailed significance level of α = 0.05, and a desired statistical power of 1 − β = 0.80, a minimum sample size of 60 participants was required. Accordingly, the enrollment of 60 participants was considered statistically sufficient to detect moderate associations between COP path length and the criterion measures.

### 2.3. Experimental Procedure

General characteristics were collected prior to balance assessment. Balance performance was evaluated using a Wii Balance Board (WBB) and the Tetrax^®^ system simultaneously. The two devices were vertically stacked, with the Tetrax^®^ system positioned on top of the WBB, allowing for concurrent measurement of postural variables. To minimize positional displacement during testing, device placement was standardized using fixed alignment markings on a wooden platform. Because the WBB recorded relative COP displacement rather than absolute force values, no additional post hoc correction for the weight of the Tetrax^®^ device was applied.

**Figure 1:**
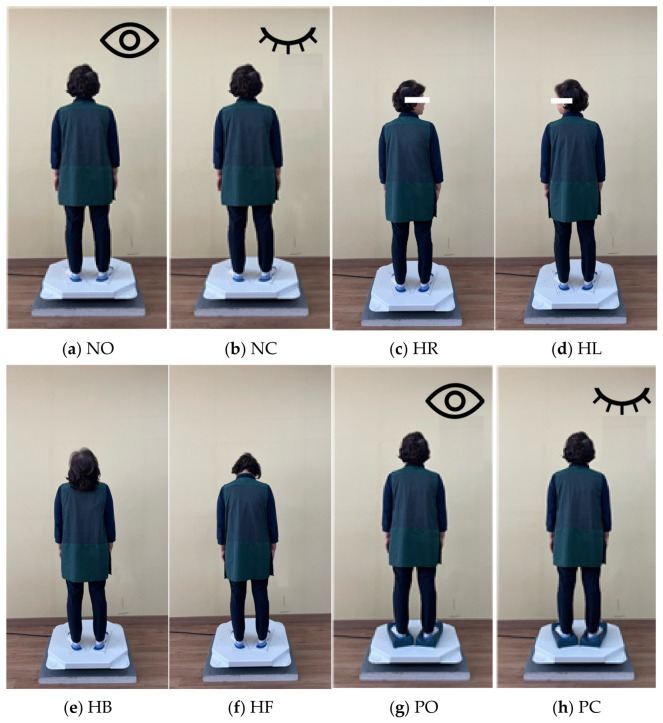
Eight measurement postures for the Tetrax static posturography system: (**a**) Normal stability, Open eyes (NO); (**b**) Normal stability, Closed eyes (NC); (**c**) Head turned Right, Closed eyes (HR); (**d**) Head turned Left, Closed eyes (HL); (**e**) Head tilted Backward, Closed eyes (HB); (**f**) Head tilted Forward, Closed eyes (HF); (**g**) Pillow, Open eyes (PO); and (**h**) Pillow, Closed eyes (PC).

Participants stood barefoot on the devices and performed simultaneous WBB and Tetrax^®^ assessments under eight standardized postural conditions according to the predefined protocol of the Tetrax^®^ system (Figure 1) [16]. These conditions systematically manipulated visual, vestibular, and somatosensory inputs to evaluate sensory integration-related balance responses. During each condition, COP path length and the Fall Index were recorded simultaneously using a test–retest protocol with two repeated measurements.

**Figure 2:**
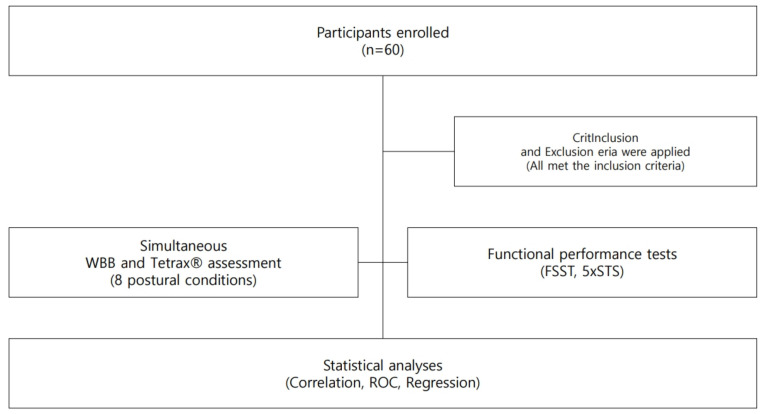
Experimental flowchart.

Following completion of the balance assessments, functional fall-risk evaluations were conducted using the FSST and 5× STS according to standardized instructions. Concurrent validity was examined by analyzing associations between COP path length and the Fall Index, whereas construct validity was assessed through correlations between COP path length and functional performance measures. ROC analysis was performed to determine optimal cut-off values for fall-risk classification. In addition, exploratory multivariable regression analyses were conducted to investigate the feasibility of developing a prototype WBB-derived fall-risk estimation model based on multiple sensory-condition COP measures. The overall experimental procedure is illustrated in Figure 2.

### 2.4. Balance Assessment

#### 2.4.1. Wii Balance Board

The Wii Balance Board (WBB) is equipped with four pressure sensors that measure body weight distribution and COP parameters [17]. COP data were collected and analyzed using Balancia software (version 2.5; Mintosys Inc., Seoul, Republic of Korea), a Windows-based posturography analysis program. The WBB was connected to a laptop computer via Bluetooth communication, and COP signals were sampled at 100 Hz. The Balancia software automatically performed signal calibration and filtering procedures during data acquisition and COP path length calculation. Shorter COP path length values indicate better postural stability. Previous studies have reported high test–retest reliability for COP path length measured using Balancia software (ICC = 0.955) [18]. In the present study, test–retest reliability ranged from ICC = 0.937 to 0.982.

#### 2.4.2. Tetrax^®^ System

The Tetrax^®^ system (Sunlight Medical Ltd., Ramat Gan, Israel) assesses postural stability and fall risk by analyzing plantar pressure distribution across four foot regions. The Fall Index was used as a reference measure of multidimensional fall-risk status. Participants performed eight test conditions: (1) Normal stability, Open eyes (NO); (2) Normal stability, Closed eyes (NC); (3) Pillow, Open eyes (PO); (4) Pillow, Closed eyes (PC); (5) Head turned Right, Closed eyes (HR); (6) Head turned Left, Closed eyes (HL); (7) Head tilted Backward, Closed eyes (HB); and (8) Head tilted Forward, Closed eyes (HF) [16]. Each condition was recorded for 32 s. The Fall Index ranges from 0 to 100, with scores of 0–57 indicating low fall risk and 58–100 indicating high fall risk.

### 2.5. Functional Performance Tests

FSST and 5× STS were administered as functional performance tests for fall-risk classification. FSST required participants to step rapidly through four squares formed by cylindrical bars in a predefined sequence, and completion time was recorded. A cut-off time of 15 s was used to classify high fall risk [5]. The 5× STS involved five consecutive sit-to-stand repetitions performed as quickly as possible from a standardized armless chair (seat height: 43 cm), with arms folded across the chest. Age-specific cut-off values were applied to classify fall risk [19]. Each functional test was performed three times to minimize measurement variability, and the mean completion time was used for analysis.

### 2.6. Statistical Analysis

Statistical analyses were conducted using SPSS Statistics (version 21.0; IBM Corp., Armonk, NY, USA) and MedCalc Software (version 20.1.4; MedCalc Software Ltd., Ostend, Belgium). Descriptive statistics were calculated as means and standard deviations. Pearson correlation coefficients were used to assess associations between COP path length, Fall Index, FSST, and 5× STS. Partial correlation analyses controlling for sex were additionally performed to account for potential sex-related influences on balance performance and fall risk.

ROC analysis was performed using the Tetrax^®^ Fall Index-based risk classification as the reference criterion to determine optimal COP path length cut-off values for fall-risk classification. Exploratory multivariable regression analyses were conducted to investigate the feasibility of developing prototype WBB-derived fall-risk estimation models based on COP path length across multiple sensory conditions. Variance inflation factors (VIFs) and tolerance statistics were additionally examined to assess multicollinearity among postural-condition variables.

The agreement and associations between regression-estimated values and reference Fall Index values were examined using paired *t*-tests, Pearson correlation coefficients, and intraclass correlation coefficients (ICCs). The significance level was set at α = 0.05.

## 3. Results

### 3.1. Participant Characteristics

Participant characteristics are summarized in Table 1. A total of 60 older adults participated in this study, including 15 men and 45 women. The mean age was 77.5 ± 8.2 years, mean height was 156.6 ± 8.4 cm, and mean body weight was 56.5 ± 10.4 kg.

### 3.2. Characteristics of Fall-Related Variables

Participants were classified into higher-risk and lower-risk groups according to the Fall Index obtained from the Tetrax^®^ system. The higher-risk group was defined as a Fall Index score of 58–100, whereas the lower-risk group was defined as a score of 0–57. Fall Index scores, COP path length values, and functional performance outcomes for both groups are presented in Table 2. Across all postural conditions, COP path length values were generally higher in the higher-risk group than in the lower-risk group. FSST and 5× STS completion times were also longer in the higher-risk group.

### 3.3. Correlation Between COP Path Length and Fall Index

Pearson correlation analysis was conducted to examine associations between COP path length and the Fall Index (Table 3). Significant positive correlations were observed across all COP path length conditions, with correlation coefficients ranging from r = 0.349 to 0.561 (*p* < 0.01). Similar correlation patterns were observed after controlling for sex using partial correlation analyses.

### 3.4. Correlation Between COP Path Length and Functional Performance Tests

Correlations between COP path length and functional performance tests are presented in Table 4. COP path length showed significant positive correlations with FSST under most conditions (r = 0.361–0.437, *p* < 0.01), with the exception of the NO condition (r = 0.247, *p* = 0.057). Similarly, 5× STS showed significant correlations under most conditions (r = 0.302–0.404, *p* < 0.05), except for NO (r = 0.190, *p* = 0.146).

### 3.5. Cut-Off Values of COP Path Length

ROC analysis was performed using the Tetrax^®^ Fall Index-based risk classification as the reference criterion to determine cut-off values for COP path length (Table 5). All postural conditions demonstrated a statistically significant ability to discriminate Tetrax^®^ Fall Index-based risk status (*p* < 0.0001). The area under the curve (AUC) values ranged from 0.783 to 0.865, indicating acceptable to good discriminatory performance. Sensitivity ranged from 66.10% to 81.36%, and specificity ranged from 70.59% to 86.27% across conditions.

**Figure 3:**
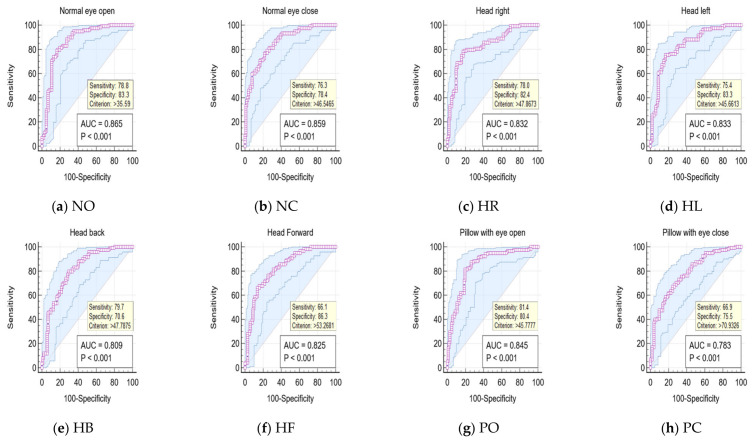
ROC curves of COP path length for Tetrax^®^ Fall-Risk classification: (**a**) Normal stability, Open eyes (NO); (**b**) Normal stability, Closed eyes (NC); (**c**) Head turned Right, Closed eyes (HR); (**d**) Head turned Left, Closed eyes (HL); (**e**) Head tilted Backward, Closed eyes (HB); (**f**) Head tilted Forward, Closed eyes (HF); (**g**) Pillow, Open eyes (PO); and (**h**) Pillow, Closed eyes (PC).

Among the evaluated conditions, the “Normal stability, Open eyes” (NO) condition demonstrated the highest discriminative performance (AUC = 0.865, 95% CI: 0.813–0.908), with a sensitivity of 78.81%, specificity of 83.33%, and the highest Youden’s Index (0.6215). ROC curves for all conditions are presented in Figure 3.

### 3.6. Regression Analysis for Predicting the Fall Index

Exploratory multivariable regression analyses were conducted to investigate the feasibility of developing WBB-derived fall-risk estimation models based on COP path length across multiple sensory postural conditions (Table 6). In the model including all eight postural conditions, the “Normal stability, Open eyes” (NO), “Head tilted Backward, Closed eyes” (HB), and “Pillow, Open eyes” (PO) conditions demonstrated statistically significant contributions, accounting for 40.7% of the variance in the reference Fall Index.

**Figure 4:**
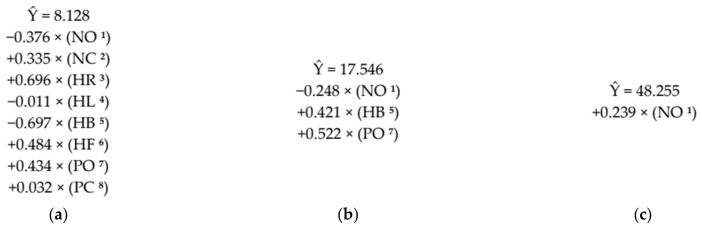
Exploratory WBB-derived regression models for estimating reference Tetrax^®^ Fall Index values from center of pressure (COP) path length measured using a Wii Balance Board: (**a**) Model including eight postural conditions; (**b**) Simplified model including three selected postural conditions; (**c**) Single-condition model using the NO posture. ^1^ Normal stability, Open eyes, ^2^ Normal stability, Closed eyes, ^3^ Head turned Right, Closed eyes, ^4^ Head turned Left, Closed eyes, ^5^ Head tilted Backward, Closed eyes, ^6^ Head tilted Forward, Closed eyes, ^7^ Pillow, Open eyes, ^8^ Pillow, Closed eyes.

The simplified three-condition model explained 30.9% of the variance, whereas the single-condition NO model explained 12.1% of the variance. Regression-based exploratory estimation equations for each model are presented in Figure 4.

Several postural-condition variables demonstrated coefficient direction reversals despite uniformly positive bivariate correlations with the Fall Index. Additional multicollinearity analysis demonstrated elevated variance inflation factor (VIF) values across multiple sensory-condition variables, suggesting substantial intercorrelation among postural conditions. Accordingly, the regression coefficients should be interpreted as statistical weighting components within exploratory multivariable estimation models rather than as isolated physiological indicators.

### 3.7. Comparison Between Regression-Estimated Values and Reference Fall Index Values

Regression-estimated values derived from the exploratory WBB-based models were compared with the reference Tetrax^®^ Fall Index values. In the eight-condition model, a statistically significant difference was observed between the estimated and reference values (t = −18.205, *p* < 0.001), although moderate correlation (r = 0.579, *p* < 0.001) and agreement (ICC = 0.658) were observed (Table 7).

The substantially elevated estimated values observed in the eight-condition model likely reflect cumulative variance inflation and scale calibration limitations associated with simultaneous inclusion of highly correlated sensory-condition COP variables. Therefore, the eight-condition model should be interpreted as an exploratory prototype estimation framework rather than a finalized clinical scoring model.

The three-condition model demonstrated no significant difference between estimated and reference values (t = −0.013, *p* = 0.990), with significant correlation (r = 0.557, *p* < 0.001) and agreement (ICC = 0.642) (Table 8). Similarly, the single-condition NO model demonstrated no significant difference (t = −0.003, *p* = 0.998), with significant but weaker correlation (r = 0.348, *p* < 0.001) and agreement (ICC = 0.356) (Table 9).

## 4. Discussion

The present study investigated the construct and concurrent validity of center of pressure (COP) path length measured using a Wii Balance Board (WBB) in relation to a clinically established posturographic fall-risk construct derived from the Tetrax^®^ system. The main findings demonstrated that WBB-derived COP path length was significantly associated with the Tetrax^®^ Fall Index as well as with functional performance measures. Furthermore, several sensory postural conditions demonstrated meaningful discriminatory performance and exploratory feasibility for approximating multidimensional fall-risk status.

Previous studies have demonstrated the feasibility of using low-cost force platforms, including the WBB, to assess postural sway and balance performance in older adults and various clinical populations [20,21,22]. However, much of the existing literature has primarily focused on reliability testing or concurrent validity with laboratory-grade force plates, rather than direct comparison with clinically established fall risk indices. In this context, the present study extends prior work by directly examining the relationship between WBB-derived COP path length and a validated fall risk index obtained from a conventional posturography system.

Correlation analyses revealed significant positive associations between COP path length and the Fall Index across all postural conditions. The strength of these relationships ranged from moderate to relatively strong, indicating that greater COP excursion is associated with higher fall risk [23,24,25]. These findings are consistent with previous research suggesting that increased postural sway and COP displacement reflect impaired balance control and elevated fall susceptibility in older adults. Notably, the magnitude of correlations observed—particularly under sensory-challenged conditions—was comparable to values reported in studies using more complex and costly posturographic systems. This suggests that COP path length measured with the WBB may be sufficiently sensitive to detect clinically relevant balance impairments.

COP path length was also significantly correlated with functional mobility and lower-extremity performance, including the Four Square Step Test (FSST) and the Five Times Sit-to-Stand test (5× STS). The observed correlation coefficients (FSST: r = 0.247–0.437; 5× STS: r = 0.190–0.404) indicate weak-to-moderate associations, consistent with prior findings demonstrating partial overlap between static posturographic measures and dynamic functional performance [26,27]. Notably, the NO condition did not reach statistical significance for either functional test (FSST: *p* = 0.057; 5× STS: *p* = 0.146), suggesting that COP path length measured under full sensory availability may be insufficient to discriminate functional performance capacity. Although modest in magnitude, such effect sizes align with previous posturographic studies examining the relationship between static sway measures and dynamic tasks, reflecting the partially overlapping yet distinct constructs of static and dynamic balance control [28,29]. While both rely on shared neuromuscular and sensory integration mechanisms, dynamic tasks additionally require anticipatory postural adjustments, transitional movements, and greater biomechanical demands. Importantly, correlations tended to be stronger under sensory-challenged conditions, such as standing on a compliant surface, suggesting that appropriately selected static tasks may better approximate functional balance demands by engaging sensory integration processes relevant to real-world mobility [30,31]. Collectively, these findings indicate that static COP measures, particularly under functionally demanding conditions, may provide complementary clinical information alongside conventional posturographic indices in fall risk assessment.

The discriminative validity of COP path length was further supported by ROC curve analysis using the Tetrax^®^ Fall Index classification as the reference criterion. All tested postural conditions demonstrated statistically significant and acceptable-to-good discriminatory performance, with area under the curve (AUC) values ranging from 0.783 to 0.865. Among these, the Normal stability, Open eyes (NO) posture exhibited the highest discriminative performance. Although some previous studies have suggested that complex or sensory-deprived conditions are necessary to optimize fall risk discrimination, the present findings indicate that a simple and time-efficient posture can yield meaningful discriminatory capability [32,33]. This may represent a practical advantage, particularly for screening in community or resource-limited settings. It should be emphasized that the ROC analyses were performed using Tetrax^®^ Fall Index classification rather than prospective fall outcomes. Therefore, the identified cut-off values reflect discrimination of an established posturographic fall-risk construct and should not be interpreted as direct predictors of future falls.

Regression analyses showed that a comprehensive model including all eight postural conditions explained 40.7% of the variance in the Fall Index. Importantly, a reduced model incorporating only three postures retained moderate explanatory power, accounting for 30.9% of the variance. Although a single-posture model demonstrated lower explanatory capacity, its simplicity and feasibility suggest potential utility in large-scale screening contexts.

It is also noteworthy that the direction of the regression coefficient for the Normal stability, Open eyes (NO) condition differed across models, appearing negative in the multi-condition models (eight-posture: B = −0.376; three-posture: B = −0.248) but positive in the single-condition model (B = +0.239). This apparent discrepancy is likely attributable to multicollinearity among the postural conditions included in the multi-condition models, which can distort individual coefficient estimates. The single-condition model, which is free from this confound, yielded a positive coefficient consistent with the theoretically expected direction whereby greater COP path length is associated with higher fall risk. This interpretation aligns with the clinical understanding that increased postural sway reflects reduced balance stability and elevated fall risk in older adults [34].

These results should be interpreted in light of the nature of the outcome variable. The Tetrax^®^ Fall Index represents an algorithm-based composite score derived from multiple posturographic parameters rather than a direct measure of prospective fall events [16]. Accordingly, the present regression findings primarily reflect the explanatory relationship between WBB-derived COP measures and an established fall risk construct. Given that fall risk in older adults is inherently multifactorial—encompassing sensory, neuromuscular, cognitive, behavioral, and environmental factors—the observed level of explained variance appears potentially meaningful within this construct-based framework [27,35]. Rather than implying complete prediction, the findings suggest that static COP measures capture components of balance-related fall susceptibility. Importantly, the elevated estimated values observed in the eight-condition model suggest that the current regression equations should not be interpreted as finalized clinical scoring systems. Rather, these findings support the preliminary feasibility of integrating multiple sensory-condition COP measures into prototype WBB-based fall-risk estimation frameworks.

When the regression-estimated values derived from WBB-based COP path length models were compared with the reference Tetrax^®^ Fall Index, both the three-condition and single-condition models demonstrated no significant differences in mean values, along with significant correlations and moderate agreement. This level of agreement is noteworthy considering the differences in hardware complexity, cost, and accessibility between the two systems. Rather than indicating direct interchangeability between devices, these findings suggest the preliminary feasibility of simplified WBB-based approaches for estimating multidimensional balance-related fall-risk constructs in community-dwelling older adults.

Several limitations should be acknowledged. First, the sample consisted of community-dwelling older adults, which may limit generalizability to individuals with neurological or musculoskeletal disorders or a history of recurrent falls. Second, the cross-sectional design and absence of prospective fall data preclude direct conclusions regarding prospective associations with future fall events. Although the present findings support the construct validity and discriminative capacity of WBB-derived COP measures, longitudinal studies incorporating actual fall incidence are required to establish prospective validity. Third, the study focused exclusively on static posturographic parameters and did not incorporate cognitive, environmental, or behavioral variables known to influence fall risk. Additionally, no formal cognitive screening was performed in the present study. Because cognitive impairment may influence postural control, sensory integration, and fall susceptibility in older adults, future studies should incorporate standardized cognitive assessments to better characterize the relationship between cognitive status and WBB-derived balance measures. Future studies integrating static COP measures with broader clinical assessments may help clarify their additional value for predicting future falls. Fourth, the concurrent measurement protocol required the Tetrax^®^ system to be positioned on top of the WBB during simultaneous data collection. Although device placement was standardized throughout testing, the additional mechanical load may have introduced minor systematic bias in COP path length estimation. Finally, the study sample consisted predominantly of female participants (75%), which may limit the generalizability of the findings to male older adults. Given the well-established sex-related differences in postural control strategies, muscle strength, and fall risk profiles, the findings should be interpreted with caution when applied to male populations. Future studies should aim for more balanced sex distributions or conduct sex-stratified analyses.

Future research should employ longitudinal designs to evaluate the prospective validity of WBB-based COP measures for actual fall events and include diverse clinical populations to enhance external validity. Furthermore, the development of simplified digital balance-screening approaches based on key postural conditions identified in this study may contribute to the advancement of accessible community-based fall-risk screening strategies in healthcare and community settings.

## 5. Conclusions

The present study demonstrated that center of pressure (COP) path length measured using a Wii Balance Board (WBB) was significantly associated with a conventional posturography-derived Fall Index and functional performance measures in community-dwelling older adults. The findings support the construct and concurrent validity of WBB-derived static posturographic measures and indicate acceptable discriminatory performance across multiple sensory postural conditions. In particular, selected postural conditions demonstrated the feasibility of approximating multidimensional balance-related fall-risk constructs derived from a clinically established posturography system. However, the regression models developed in this study should be interpreted as preliminary exploratory frameworks rather than finalized clinical scoring systems. Given the low cost, portability, and accessibility of the WBB, these findings support the potential utility of simplified WBB-based balance assessment approaches in community and clinical environments. Further longitudinal studies incorporating prospective fall outcomes and diverse clinical populations are required to establish prospective validity and broader external applicability.

## Figures and Tables

**Table 1:** Participants’ characteristics (*n* = 60).

	M ± SD ^1^
Sex (male/female)	15/45
Age (years)	77.5 ± 8.2
Height (cm)	156.6 ± 8.4
Weight (kg)	56.5 ± 10.4

^1^ Mean ± Standard deviation.

**Table 2:** Variables Related to Participants’ Fall Index (*n* = 60).

Test	M ± SD ^1^	
	Higher-Risk Group (n = 33)	Lower-Risk Group (n = 27)
Fall index (score)	84.46 ± 15.07	30.85 ± 13.90
COP path length ^2^ (cm)		
(a) NO	54.75 ± 57.66	29.80 ± 8.46
(b) NC	65.32 ± 33.61	39.61 ± 10.95
(c) HR	62.59 ± 26.03	40.00 ± 12.12
(d) HL	64.98 ± 35.06	40.09 ± 15.11
(e) HB	66.17 ± 30.61	44.11 ± 17.05
(f) HF	64.56 ± 31.74	41.70 ± 15.18
(g) PO	65.17 ± 32.45	41.59 ± 13.03
(h) PC	88.02 ± 31.99	60.65 ± 20.69
Physical function		
FSST ^3^ (s)	17.33 ± 3.02	13.98 ± 2.78
5× STS ^4^ (s)	19.77 ± 5.97	14.69 ± 2.57

^1^ Mean ± Standard deviation. ^2^ WBB COP path length: (a) Normal stability, Open eyes (NO); (b) Normal stability, Closed eyes (NC); (c) Head turned Right, Closed eyes (HR); (d) Head turned Left, Closed eyes (HL); (e) Head tilted Backward, Closed eyes (HB); (f) Head tilted Forward, Closed eyes (HF); (g) Pillow, Open eyes (PO); and (h) Pillow, Closed eyes (PC). ^3^ Four Square Step Test. ^4^ Five Times Sit to Stand test.

**Table 3:** Correlation between COP Path Length and Tetrax^®^ Fall Index (n = 60).

COP Path Length ^1^ (cm)	*r* (*p*)
(a) NO	0.349 (0.006)
(b) NC	0.533 (<0.001)
(c) HR	0.561 (<0.001)
(d) HL	0.511 (<0.001)
(e) HB	0.507 (<0.001)
(f) HF	0.526 (<0.001)
(g) PO	0.507 (<0.001)
(h) PC	0.515 (<0.001)

^1^ WBB COP path length: (a) Normal stability, Open eyes (NO); (b) Normal stability, Closed eyes (NC); (c) Head turned Right, Closed eyes (HR); (d) Head turned Left, Closed eyes (HL); (e) Head tilted Backward, Closed eyes (HB); (f) Head tilted Forward, Closed eyes (HF); (g) Pillow, Open eyes (PO); and (h) Pillow, Closed eyes (PC).

**Table 4:** Correlations between COP Path Length and Functional Performance Measures (n = 60).

	*r* (*p*)	
COP Path Length ^1^ (cm)	FSST ^2^ (s)	5× STS ^3^ (s)
(a) NO	0.247 (0.057)	0.190 (0.146)
(b) NC	0.394 (0.002)	0.366 (0.004)
(c) HR	0.415 (0.001)	0.404 (0.001)
(d) HL	0.383 (0.003)	0.328 (0.011)
(e) HB	0.361 (0.005)	0.302 (0.019)
(f) HF	0.417 (0.001)	0.307 (0.017)
(g) PO	0.437 (<0.001)	0.330 (0.010)
(h) PC	0.406 (0.001)	0.385 (0.002)

^1^ WBB COP path length: (a) Normal stability, Open eyes (NO); (b) Normal stability, Closed eyes (NC); (c) Head turned Right, Closed eyes (HR); (d) Head turned Left, Closed eyes (HL); (e) Head tilted Backward, Closed eyes (HB); (f) Head tilted Forward, Closed eyes (HF); (g) Pillow, Open eyes (PO); and (h) Pillow, Closed eyes (PC). ^2^ Four Square Step Test. ^3^ Five Times Sit to Stand test.

**Table 5:** ROC Analysis of COP Path Length for Tetrax^®^ Fall-Risk Classification (n = 60).

COP Path Length ^1^ (cm)	AUC (95% CI)	*p*-Value	Sensitivity (%)	Specificity (%)	Youden’s Index
(a) NO	0.865 (0.813–0.908)	<0.0001	78.81	83.33	0.6215
(b) NC	0.859 (0.805–0.902)	<0.0001	76.27	78.43	0.5470
(c) HR	0.832 (0.776–0.879)	<0.0001	77.97	82.35	0.6032
(d) HL	0.833 (0.777–0.880)	<0.0001	75.42	83.33	0.5876
(e) HB	0.809 (0.751–0.859)	<0.0001	79.66	70.59	0.5025
(f) HF	0.825 (0.768–0.872)	<0.0001	66.10	86.27	0.5238
(g) PO	0.845 (0.791–0.890)	<0.0001	81.36	80.39	0.6175
(h) PC	0.783 (0.723–0.836)	<0.0001	66.95	75.49	0.4244

^1^ WBB COP path length: (a) Normal stability, Open eyes (NO); (b) Normal stability, Closed eyes (NC); (c) Head turned Right, Closed eyes (HR); (d) Head turned Left, Closed eyes (HL); (e) Head tilted Backward, Closed eyes (HB); (f) Head tilted Forward, Closed eyes (HF); (g) Pillow, Open eyes (PO); and (h) Pillow, Closed eyes (PC).

**Table 6:** Exploratory Regression Models for Estimating Tetrax^®^ Fall Index from WBB-Derived COP Path Length (n = 60).

	Eight Postures		Three Postures		Normal Stability, Open Eyes (NO)	
	B	t	B	t	B	t
Fall index	8.128	1.142	17.546	2.630	48.255	12.626
COP path length						
(a) NO	−0.376	−2.878 **	−0.248	−2.324 *	0.239	3.859 **
(b) NC	0.335	1.267				
(c) HR	0.696	1.887				
(d) HL	−0.011	−0.033				
(e) HB	−0.697	−2.007 *	0.421	2.198 *		
(f) HF	0.484	1.688				
(g) PO	0.434	2.121 *	0.522	2.532 *		
(h) PC	0.032	0.216				
F	8.649 **		15.804 **		14.934 **	
Adjusted R^2^	0.407		0.309		0.121	
R^2^ change	0.360		0.289		0.113	

Regression coefficients should be interpreted within the context of exploratory multivariable estimation models because of intercorrelations among sensory-condition variables. (a) Normal stability, Open eyes (NO); (b) Normal stability, Closed eyes (NC); (c) Head turned Right, Closed eyes (HR); (d) Head turned Left, Closed eyes (HL); (e) Head tilted Backward, Closed eyes (HB); (f) Head tilted Forward, Closed eyes (HF); (g) Pillow, Open eyes (PO); and (h) Pillow, Closed eyes (PC). *: *p* < 0.05, **: *p* < 0.01.

**Table 7:** Comparison between Exploratory Eight-Condition Regression-Estimated Values and Reference Tetrax^®^ Fall Index (n = 60).

	Tetrax Fall Index	Regression-Estimated Values ^a^	t ^b^ (*p*)	r ^c^ (*p*)	ICC (95% CI)
Fall index ^d^	58.58 ± 30.38	136.32 ± 54.89	−18.205 (<0.001)	0.579 (<0.001)	0.658 (0.501–0.766)

Estimated values exceeding the original Tetrax^®^ Fall Index range likely reflect exploratory model scaling limitations associated with multicollinearity among sensory-condition variables. ^a^ Regression-estimated values derived from WBB-based COP path length models. ^b^ Results of paired-samples *t*-test. ^c^ Results of Pearson correlation analysis. ^d^ Reference Fall Index obtained from the Tetrax^®^ system.

**Table 8:** Comparison between Three-Condition Regression-Estimated Values and Reference Tetrax^®^ Fall Index (n = 60).

	Tetrax Fall Index	Regression-Estimated Values ^a^	t ^b^ (*p*)	r ^c^ (*p*)	ICC (95% CI)
Fall index ^d^	58.58 ± 30.38	58.61 ± 16.91	−0.013 (0.990)	0.557 (<0.001)	0.642 (0.479–0.755)

^a^ Regression-estimated values derived from WBB-based COP path length models. ^b^ Results of paired-samples *t*-test. ^c^ Results of Pearson correlation analysis. ^d^ Reference Fall Index obtained from the Tetrax^®^ system.

**Table 9:** Comparison between Single-Condition Regression-Estimated Values and Reference Tetrax^®^ Fall Index (n = 60).

	Tetrax Fall Index	Regression-Estimated Values ^a^	t ^b^ (*p*)	r ^c^ (*p*)	ICC (95% CI)
Fall index ^d^	58.58 ± 30.38	58.58 ± 10.58	−0.003 (0.998)	0.348 (<0.001)	0.356 (0.060–0.558)

^a^ Regression-estimated values derived from WBB-based COP path length models. ^b^ Results of paired-samples *t*-test. ^c^ Results of Pearson correlation analysis. ^d^ Reference Fall Index obtained from the Tetrax^®^ system.

## Data Availability

The data presented in this study are available from the corresponding author upon reasonable request.
